# Monogenic Neonatal Diabetes: Clinical Presentations, Genetic Findings, and Response to Therapy in a Retrospective Case Series

**DOI:** 10.7759/cureus.102578

**Published:** 2026-01-29

**Authors:** Fatimazahra Yakine, Ilham Bouarab, Fatima Zahra Alaoui-Inboui, Meryem Ech-charafi, Houda Benmohamed, Farida Jennane, Bouchra Slaoui

**Affiliations:** 1 Pediatrics Department 2, Pediatrics Endocrinology and Diabetology Unit, Abderrahim Harouchi Mother-Child Hospital, Casablanca, MAR; 2 Pediatrics Department 2, Pediatric Endocrinology and Diabetology Unit, Abderrahim Harouchi Mother-Child Hospital, Casablanca, MAR; 3 Pediatrics Department 2, Pulmonology and Allergology Unit, Abderrahim Harouchi Mother-Child Hospital, Casablanca, MAR

**Keywords:** abcc8 mutation, beta cell, hypoglycemic sulfonylureas, insulin therapy, neonatal diabetes

## Abstract

Introduction

Monogenic neonatal diabetes mellitus (NDM) is a rare form of diabetes, presenting within the first six months of life and caused by pathogenic variants affecting pancreatic β-cell development or function. Because its initial presentation may overlap with type 1 diabetes, molecular diagnosis is crucial, as it directly influences prognosis and treatment - particularly the potential responsiveness to sulfonylureas in ATP-sensitive potassium (KATP)-channel-related NDM. This study reports a retrospective descriptive case series and aims to characterize the clinical and genetic features of infants with NDM, to improve therapeutic management and long-term outcomes.

Materials and methods

We conducted a retrospective descriptive case series of infants diagnosed with diabetes before six months of age, hospitalized in the Pediatric Endocrinology Unit of the Abderrahim Harouchi Mother-Child Hospital, Casablanca, Morocco, between January 2018 and December 2025. Clinical presentation, biochemical data, insulin requirements, genetic results, and outcomes were extracted from medical records. Genetic testing was performed through next-generation sequencing (NGS), or targeted Sanger sequencing when financially feasible.

Results

Ten infants were included (nine males and one female), with a mean age at diagnosis of 71 days. Diabetic ketoacidosis (DKA) was the presenting feature in all cases. Consanguinity was reported in 55% of families. Pathogenic or likely pathogenic variants were identified in six infants (60%), involving ABCC8, INS, EIF2AK3, CASP10, and chromosome 6q23-24 duplication, including two syndromic forms. Two infants with ABCC8 mutations achieved insulin independence with sulfonylurea therapy. Syndromic etiologies - Wolcott-Rallison syndrome, Donohue syndrome, and autoimmune lymphoproliferative syndrome type IIA (ALPS-type IIA) - were associated with severe multisystemic involvement. Three children had no identifiable pathogenic variant, despite clinical features consistent with NDM. Long-term outcomes varied widely, ranging from normal neurodevelopment to early mortality in Donohue syndrome.

Conclusion

This retrospective descriptive case series highlights the marked genetic heterogeneity and clinical variability of neonatal diabetes in a resource-limited setting. Genetic testing enabled precision therapy in infants harboring KATP-channel mutations and clarified prognosis in syndromic forms. Expanding access to molecular diagnostics remains essential to improve equity in care, optimize metabolic outcomes, and support individualized management strategies.

## Introduction

Neonatal diabetes mellitus (NDM) is a monogenic disorder characterized by the onset of persistent hyperglycemia within the first six months of life. It is a rare but severe condition, with an estimated incidence ranging from approximately 1 in 90,000 to 1 in 160,000 live births in contemporary cohorts [1]. NDM results from defects in genes involved in pancreatic β-cell development, insulin synthesis, or the regulation of insulin secretion, most commonly affecting the ATP-sensitive potassium (KATP) channel genes KCNJ11 and ABCC8, as well as the proinsulin gene (INS), glucokinase (GCK), and transcription factors essential for pancreatic morphogenesis. Other presentations reflect multisystem disorders, including Wolcott-Rallison syndrome and Donohue syndrome, as well as imprinted loci, such as chromosome 6q24 [2]. Many affected infants are born small for gestational age, reflecting impaired prenatal insulin secretion, as insulin plays a critical role in fetal growth and anabolic processes during intrauterine development. Prompt recognition and early management are therefore essential to prevent acute metabolic decompensation and to optimize growth and neurodevelopmental outcomes [3].

Two major clinical subtypes of NDM have been identified, based primarily on the duration of insulin dependency: permanent neonatal diabetes mellitus (PNDM) and transient neonatal diabetes mellitus (TNDM). PNDM represents the chronic form and requires lifelong insulin therapy due to a persistent defect in β-cell function. In contrast, TNDM accounts for approximately 50%-60% of all NDM cases [4,5]. This form is characterized by spontaneous remission, typically occurring within the first year of life; however, affected individuals remain at risk of metabolic instability throughout life. Recurrence of diabetes is common, often emerging during late childhood, adolescence, or adulthood. These relapses usually resemble non-autoimmune type 1 diabetes, though the underlying mechanisms - whether persistent insulin deficiency, acquired insulin resistance, or a combination of both - remain subjects of ongoing investigation [5].

The identification of the molecular basis of NDM has profoundly transformed clinical management by enabling genotype-driven therapeutic strategies. In particular, treatment with sulfonylureas - especially glibenclamide - is recommended for NDM caused by KCNJ11 and ABCC8 mutations, and represents a major shift away from insulin therapy. Beyond achieving excellent glycemic control, glibenclamide has been shown to significantly improve neurological and neuropsychological outcomes in affected individuals, with earlier initiation of therapy associated with greater developmental benefit [3]. In contrast, infants with syndromic or recessive forms of NDM often experience poorer outcomes due to associated hepatic, skeletal, or immune dysfunction. Despite major advances in genetic characterization, access to molecular testing remains limited in many low- and middle-income countries, contributing to diagnostic delays and suboptimal management. In our setting, the heterogeneity of clinical presentations and the variable availability of genetic analysis pose significant challenges to standardized care.

This retrospective descriptive case series aimed to characterize the clinical, biochemical, and genetic features of infants diagnosed with neonatal diabetes in our tertiary center over an eight-year period, and to evaluate their therapeutic trajectories and long-term outcomes, with particular emphasis on the impact of genetic testing on individualized management. The primary objective was phenotypic and genotypic characterization, rather than comparative analysis.

## Materials and methods

We conducted a retrospective, descriptive case series, including newborns and infants diagnosed with neonatal diabetes, and followed at the Endocrinology Unit of the Abderrahim Harrouchi Mother and Child Hospital, Ibn Rochd University Hospital Center, Casablanca, Morocco. The study covered an eight-year period, from January 2018 to December 2025, and aimed to characterize clinical presentations, biochemical profiles, genetic findings, therapeutic approaches, and outcomes.

A total of 10 cases of neonatal diabetes were included. Diagnosis of NDM was established based on a combination of clinical, biochemical, and immunological criteria, supplemented by genetic confirmation when available. The initial clinical presentation, most frequently diabetic ketoacidosis (DKA) or persistent hyperglycemia, was evaluated at admission. Hyperglycemia, resolving with insulin therapy in the absence of anti-pancreatic islet (anti-GAD, anti-IA2, or anti-ZnT8) antibodies, supported the suspicion of a monogenic form. Genetic testing was performed in 8 of the 10 patients, with pathogenic or likely pathogenic variants identified in six cases. All molecular analyses were carried out at the Necker Enfants Malades Hospital, Paris, France.

Inclusion and exclusion criteria

We included all children younger than six months at diagnosis, presenting with persistent hyperglycemia greater than 2 g/L (11.1 mmol/L) on at least two measurements, and with complete clinical and biochemical records. We excluded medical records with missing essential data, patients whose hyperglycemia was diagnosed after six months of age, and infants with transient, stress-induced hyperglycemia related to acute illness or prematurity.

Data collection

Medical records were retrieved from the hospital archive system and reviewed by the investigators. Relevant information was extracted and entered into a dedicated electronic database, created using Microsoft Excel (Microsoft® Corp., Redmond, WA, USA). Data collection covered epidemiological, clinical, biochemical, and genetic variables. Only one newborn in the cohort underwent prenatal or immediate neonatal screening, performed in a private setting, owing to a positive family history of transient neonatal diabetes. All other cases were diagnosed following the onset of clinical symptoms, such as dehydration, poor feeding, failure to thrive, or DKA. Medical records were systematically reviewed to extract demographic information (sex, gestational age, and birth weight), family history of diabetes, consanguinity, clinical presentation at diagnosis, and presence of comorbidities or syndromic features. Growth parameters were expressed as standard deviation scores (SDS), according to the WHO growth charts. Biochemical data at admission and during follow-up included blood glucose, HbA1c, C-peptide, insulin levels, electrolytes, liver and renal function tests, inflammatory markers (CRP and procalcitonin), and autoantibody profiles (anti-GAD, anti-IA2, and anti-insulin).

Treatment protocols

All patients presenting with DKA were initially managed according to standard pediatric protocols, including intravenous insulin infusion (0.025-0.05 IU/kg/h) and correction of fluid and electrolyte imbalances. After stabilization, insulin therapy was transitioned to subcutaneous regimens - either basal-bolus, twice-daily injections, or continuous subcutaneous insulin infusion (insulin pump) - based on age, weight, and severity of diabetes. For infants harboring KATP-channel mutations, sulfonylurea therapy was initiated using standardized protocols under inpatient supervision, with gradual withdrawal of insulin and titration of oral therapy, according to capillary glucose monitoring.

Genetic analysis

Genetic testing was performed when feasible. Sequencing approaches included targeted Sanger sequencing for known NDM-associated genes (ABCC8, KCNJ11, and INS) and next-generation sequencing (NGS) panels, covering all reported monogenic diabetes genes. Chromosome 6q24 abnormalities were assessed using array comparative genomic hybridization or multiplex ligation-dependent probe amplification (MLPA). 

Ethical considerations

This retrospective study was conducted in accordance with institutional ethical standards. Patient confidentiality was maintained by anonymizing all data during extraction and database entry.

Outcome definitions

Clinical outcomes were evaluated using predefined operational criteria: (i) good glycemic control: HbA1c <7.5% without recurrent severe hypoglycemia or DKA; (ii) poor glycemic control: HbA1c ≥7.5% and/or recurrent DKA or severe hypoglycemia; (iii) insulin independence: cessation of insulin ≥3 months without ketosis or severe hyperglycemia; (iv) normal neurodevelopment: age-appropriate milestones documented by neurology or standardized developmental assessments; (v) growth delay: height and/or weight <-2 SD based on the WHO standards. Therapeutic outcomes were assessed at the last available follow-up.

Statistical analysis

Descriptive statistics were used to summarize clinical, biochemical, and genetic data. Continuous variables are reported as mean ± standard deviation or median (range), and categorical variables as counts and percentages. Due to the small sample size, formal hypothesis testing was not performed.

## Results

A total of 10 patients were included. The mean age at diagnosis was 71 days (range: 31-150 days), with a marked male predominance (nine males and one female). Parental consanguinity was present in 55% of cases, and a family history of type 2 diabetes mellitus was reported in two infants. All patients presented with DKA at diagnosis.

Clinical presentation

Neurological symptoms were observed in 33% of infants (confusion in three cases and hypotonia in one case), including one case complicated by sepsis. Respiratory or gastrointestinal manifestations were present in four neonates. Three infants exhibited progressive dehydration and poor weight gain from birth. Abdominal ultrasonography was normal in all patients, with no evidence of pancreatic agenesis.

Initial management

All infants received continuous intravenous insulin infusion (0.025 IU/kg/h) until resolution of neurological, respiratory, and digestive symptoms, normalization of capillary glucose, and clearance of urinary ketones. Fluid and electrolyte monitoring was performed at baseline (H0) and at H2, H6, H12, and H24. After stabilization, two infants were transitioned to twice-daily insulin injections, seven to a basal-bolus regimen, and one to continuous subcutaneous insulin infusion. Glycemic control was particularly challenging in the two syndromic cases (Wolcott-Rallison syndrome and Donohue syndrome).

Autoimmunity and genetics

Anti-islet autoantibodies were assessed in eight patients and were negative in all tested cases. Genetic testing identified: (i) ABCC8 mutations in two infants (20%), both successfully transitioned to sulfonylurea therapy; (ii) 6q23-24 duplication consistent with transient neonatal diabetes in one infant; (iii) INS mutation in one infant with severe neurodevelopmental delay; (iv) EIF2AK3 mutation confirming Wolcott-Rallison syndrome in one infant; (v) no identifiable pathogenic variant in three infants; (vi) genetic testing not performed in two infants due to resource limitations.

Outcomes

Two infants with ABCC8 mutations achieved complete insulin independence under glibenclamide. One infant with transient neonatal diabetes experienced spontaneous remission. Syndromic cases (Wolcott-Rallison and Donohue syndromes) exhibited severe metabolic instability and multisystem involvement; the infant with Donohue syndrome died at four months from sepsis.

At last follow-up (median age: 4.5 years), seven children remained on insulin therapy with variable glycemic control (HbA1c range: 6.7%-8%). Growth impairment was noted in syndromic and autoimmune cases. One infant was diagnosed with autoimmune lymphoproliferative syndrome (ALPS) (CASP10 mutation) after persistent cytopenias and hepatic dysfunction.

Detailed clinical, biochemical, therapeutic, genetic, and developmental characteristics are summarized in Tables 1-2.

**Table 1:** Comparison of clinical characteristics of infants with neonatal diabetes mutations

​	ABCC8​		Duplication of chromosome region 6q23-24	Insulin gene ​	EIF2AK3 gene ​	CASP10
Number (%)		2 (10)​	1 (10)	1 (10)​	1 (10)​	1 (10)
Age at diagnosis (months)		2.2​	1	1.77	4.28​	4.33
Gestational age (weeks)		39.2​	38	38.8​	38.2​	39.3
Birth weight (grams)		4100​	2080	1950​	2500​	6700
Birth weight <3rd percentile		1​	1	1​	0​	0
Diabetic ketoacidosis		2	1	1​	1​	1

**Table 2:** Biological and therapeutic characteristics of patients at last visit

Patient	Current age (years)	Insulin requirement (IU/kg/day)	Sulfonylurea dose (mg/kg/day)	HbA1c (%)	Mutation
1​	4	-​	0.4	6.4	ABCC8
2​	12.25	0.8	-​	7.6	ABCC8
3	0.41	0	-	5.4	Duplication of chromosome 6q23-24
4	18	0.6	-​	6.7	Unknown
5	4.58	0.8	-​	7.2	CASP10
6	7.83	0.8	-​	8	INS mutation
7	1.16	-​	-​	-​	EIF21K3
8	Deceased	-​	-​	-​	Unknown
9	5.41	0.8	-​	7.6	Unknown
10	3.25	1	-	7.5	Unknown

Patient 1 was a male infant born at term with an appropriate birth weight and no intrauterine growth restriction (IUGR), to first-degree consanguineous parents. He was admitted at 2 months and 10 days of age for DKA. After initial intravenous insulin therapy, he was transitioned to a basal-bolus regimen with low insulin requirements (0.2 IU/kg/day). Anti-islet autoantibodies were negative.

Direct sequencing of the KATP channel genes identified a pathogenic variant in ABCC8, affecting the SUR1 subunit. At 19 months of age, a supervised inpatient transition to sulfonylurea therapy (glibenclamide) was initiated according to a standardized protocol. Glibenclamide was started at 0.1 mg/kg every 12 hours, accompanied by a 50% reduction in prandial insulin, while maintaining the basal dose. Insulin boluses were subsequently discontinued, and the basal rate was progressively tapered. Sulfonylurea dosing was titrated based on capillary glucose monitoring. Complete insulin withdrawal was achieved within five days.

At four years and six months of age, the patient remains on glibenclamide (0.4 mg/kg/day) with excellent glycemic control (mean HbA1c, 6.2%). Neurodevelopment is normal, and no adverse effects have been observed.

Patient 2 was a male infant born at term to second-degree consanguineous parents, without IUGR. He presented with DKA at two months and two days of age, initially managed with intravenous insulin, followed by subcutaneous insulin therapy (0.5 IU/kg/day). Anti-islet autoantibodies were negative.

Genetic analysis, via direct sequencing of the KATP channel genes, revealed a pathogenic ABCC8 (SUR1) mutation. At 22 months, insulin was discontinued by the parents due to sustained normoglycemia. One month later, glibenclamide was introduced, resulting in good glycemic control. At four years and one month, neurodevelopment was normal, and HbA1c was 6.7%. After a two-year loss to follow-up and treatment discontinuation, he was readmitted with poor glycemic control (HbA1c, 14%) and is currently managed with a basal-bolus regimen (HbA1c, 7.6%).

Patient 3 was a male neonate with low birth weight (2080 g) and macroglossia, born to non-consanguineous parents. Two paternal cousins had a history of transient neonatal diabetes. He presented at 29 days of life with mild DKA and was initially treated with intravenous insulin, followed by insulin detemir (0.2 IU/kg/day), which was discontinued after one month. Anti-islet autoantibodies were negative. NGS identified a paternally inherited duplication of chromosome 6q23-24, consistent with TNDM.

Patient 4 was a male infant born at term to consanguineous parents, with a family history of TNDM and paternal type 2 diabetes. He presented with DKA at four months and five days of age. Insulin therapy was discontinued at 20 months due to recurrent hypoglycemia. At 10 years and 3 months, hyperglycemia recurred, requiring reintroduction of insulin (0.6 IU/kg/day, HbA1c 7.5%). Genetic testing was not performed.

Patient 5 was a male infant from a non-consanguineous union who developed severe DKA at four months. Pregnancy and birth history were unremarkable, with no evidence of IUGR. After initial stabilization with intravenous insulin, the patient was transitioned to subcutaneous insulin therapy.

By six months of age, insulin requirements remained elevated (1.0-1.2 IU/kg/day). The patient developed chronic diarrhea beginning at four months, associated with growth faltering affecting both weight and length. Glycemic control remained suboptimal, with HbA1c persistently between 9% and 10%, largely due to psychosocial family constraints and poor treatment adherence. No episodes of severe hypoglycemia or recurrent ketoacidosis were documented. Further investigations revealed elevated anti-tissue transglutaminase IgA antibodies (98 U/mL). Upper gastrointestinal endoscopy demonstrated subtotal villous atrophy (Marsh grade III-IV). A gluten-free diet was initiated; however, no clinical improvement was observed. Fasting insulin and C-peptide levels were low, and pancreatic autoantibodies (anti-GAD, anti-IA2, and insulin autoantibodies) were negative. Comprehensive genetic screening did not identify any known pathogenic variants associated with neonatal diabetes.

At 11 months, he was readmitted with severe DKA, complicated by sepsis. Clinical examination revealed pallor, fever (39.8°C), achromic maculopapular lesions, oral thrush, and febrile splenomegaly (10 cm). The association of insulin-dependent diabetes, enteropathy, dermatological manifestations, and male sex raised suspicion for immune dysregulation polyendocrinopathy enteropathy X-linked (IPEX) syndrome. Paraclinical investigations showed bicytopenia (anemia 8.2 g/dL; thrombocytopenia 71,000/mm³), elevated transaminases (AST 152 U/L and ALT 117 U/L), ferritin 115 ng/mL, procalcitonin 5.57 ng/mL, and CRP 90 mg/L. Bone marrow aspirate was normal, and the Coombs test was negative. Immunoglobulin levels and lymphocyte subsets were within normal limits. Management included 10 days of broad-spectrum antibiotics, four days of intravenous insulin, three days of intravenous immunoglobulins, transfusion of packed red blood cells and platelets, and three months of oral corticosteroids, with transient improvement, followed by transition to insulin pump therapy.

At 13 months, the child still exhibited persistent cytopenias and hepatic dysfunction, with transaminase levels 10-fold above normal. Autoimmune serologies were positive (anti-smooth muscle, antinuclear, and anti-LKM antibodies). Genetic testing ultimately identified ALPS type IIA (CASP10 mutation), involving defective lymphocyte apoptosis.

He is currently four years and seven months old, on insulin pump therapy (0.8 IU/kg/day), with satisfactory glycemic control (HbA1c 7.2%; TIR 73% without severe hypoglycemia). Growth parameters are stable (-2.2 SD). After failure of corticosteroids for autoimmune hepatitis, multiple immunosuppressive therapies were initiated: first azathioprine, followed by mycophenolate mofetil (600 mg/m²/day).

Patient 6 was a male infant born at term with severe symmetric IUGR (birth weight 1950 g; length 44 cm; head circumference 33.5 cm), who presented with DKA at 53 days of age. C-peptide and insulin levels were markedly low. Autoantibodies (anti-islet, anti-insulin, anti-IA2, anti-GAD) were negative. Genetic testing revealed a pathogenic INS mutation. He developed severe neurodevelopmental delay, with persistent hypotonia and the inability to sit or walk by age 3 years and 10 months. He remains on basal-bolus insulin (0.8 IU/kg/day; HbA1c 8%).

Patient 7 was a male neonate born at term from a second-degree consanguineous union, with IUGR, who developed DKA following septic shock at 30 days of age. Clinical examination showed no facial dysmorphism, hepatosplenomegaly, or skeletal abnormalities. Paraclinical investigations revealed normal renal and hepatic function, no anemia or neutropenia, low C-peptide, and negative autoantibodies. He was managed with a basal-bolus insulin regimen. Genetic testing identified a homozygous EIF2AK3 class 5 pathogenic variant, confirming Wolcott-Rallison syndrome.

At one year and five months, he showed growth delay (-2 SD) without evidence of bone dysplasia or gastrointestinal/hepatic involvement. At two years and three months, he developed cholestatic jaundice, edema, and oliguria, with severe hepatic cytolysis (AST/ALT 5280 U/L), reduced prothrombin time (23%), and acute kidney injury (creatinine 8.9 mg/L, urea 0.46 g/L, GFR 30 mL/min). Hepatitis A, Epstein-Barr virus (EBV), and cytomegalovirus (CMV) serologies were negative, but herpes simplex virus (HSV) IgM was positive. He received acyclovir (30 mg/kg/day for 21 days) and intravenous corticosteroids (1 mg/kg/day for five days), with clinical improvement.

Patient 8 was a male newborn who presented at 35 days of age with DKA and severe dysmorphism. He was born to second-degree consanguineous parents at term, with IUGR (birth weight 2000 g), and had previously been hospitalized at day 20 for sepsis with enterocolitis. Examination showed hypotonia and severe growth retardation (-3 SD), with marked dysmorphism, including exophthalmos, thick lips, wide nasal wings, large ears, dense hair, melanoderma, neck desquamation, hirsutism, distended abdomen, and bilateral hydrocele with genital hypertrophy. Laboratory tests revealed a blood glucose of 4 g/L and normal 17-hydroxyprogesterone. Adrenal insufficiency was investigated due to melanoderma and genital enlargement. The constellation of findings suggested Donohue syndrome (leprechaunism). Abdominal ultrasound showed hepatomegaly, and echocardiography was normal. Genetic testing targeting the INSR gene was initiated. The infant required high-dose insulin (2 IU/kg/day). He died at four months from severe sepsis.

Patient 9 was a female infant born at term to non-consanguineous parents, who presented with DKA at three months of age. Antibody testing could not be performed for financial reasons. Genetic testing did not identify pathogenic variants. She remains stable at five years and five months on standard subcutaneous insulin (0.7 IU/kg/day; HbA1c 7.4%).

Patient 10 was a male infant born at term to first-degree consanguineous parents, who presented with DKA at 40 days of age. Genetic testing was not performed due to a lack of resources. He remains stable at three years and three months on basal-bolus insulin (0.7 IU/kg/day; HbA1c 7.5%).

## Discussion

Our series of 10 cases of NDM illustrates the marked phenotypic and genotypic heterogeneity of this rare monogenic condition, in keeping with observations from international cohorts. NDM may present as either TNDM, accounting for approximately 50%-60% of cases, or PNDM [4,5]. In contrast to published epidemiological data, only one case of transient diabetes was identified in our cohort, underscoring the variability in clinical presentation, as well as the potential influence of referral patterns, diagnostic delays, and population-specific genetic backgrounds.

Multiple genetic mechanisms contribute to the development of NDM. The most frequent include chromosome 6q24 abnormalities responsible for TNDM, such as paternal uniparental disomy, partial duplication, or methylation defects at the PLAGL1/ZAC locus; mutations affecting the KATP channel subunits (KCNJ11 ~30%, ABCC8 ~19%); INS gene mutations (~20%) impairing insulin synthesis or β-cell survival; GCK mutations (~4%) leading to GCK-related NDM; and more rarely, PDX1 mutations (<1%) affecting pancreatic development [6].

In line with established cohorts, our series demonstrated substantial genetic diversity, with pathogenic variants involving KCNJ11, chromosomal imbalance encompassing 6q24, INS mutations, and syndromic etiologies such as Wolcott-Rallison and Donohue syndromes. These findings are consistent with current International Society for Pediatric and Adolescent Diabetes (ISPAD) Clinical Practice Consensus Guidelines, which describe a wide spectrum of genetic mechanisms ranging from imprinting abnormalities at 6q24 to defects in KATP channel subunits (KCNJ11 and ABCC8), the INS gene, GCK, and transcription factors critical for pancreatic development. Collectively, these data underscore the necessity of comprehensive molecular genetic testing in all infants presenting with diabetes before six months of age [3].

The mean age at diagnosis in our cohort (71 days) was notably later than that reported in many published series, where diagnosis often occurs within the first week of life, particularly in transient forms associated with 6q24 abnormalities. This discrepancy likely reflects delayed presentation, limited access to specialized neonatal and genetic services, and the contribution of syndromic or recessive mutations that may manifest later in infancy.

Abnormalities involving chromosome 6q24 represent the most frequent cause of TNDM. These cases are typically sporadic, although a positive family history is reported in approximately one-third of patients [4,7]. Congenital malformations, particularly involving the cardiac, renal, or urinary systems, may be present [8]. While IUGR is a consistent clinical feature, disease permanence cannot be reliably predicted based solely on phenotype [9,10]. Therefore, parents of children with TNDM should internalize the high risk of their child's future diabetes relapse, and these children may benefit from annual HbA1c testing [3].

Mutations in KCNJ11 and ABCC8 represent the most common cause of PNDM. The KATP channel plays a central role in coupling glucose metabolism to insulin secretion, whereby increased intracellular ATP closes the channel, leading to membrane depolarization, calcium influx, and insulin exocytosis [11,12]. Activating mutations impair channel closure and insulin release, while expression of KATP channels in the central nervous system may explain associated neurological and cognitive abnormalities [3]. Oral sulfonylureas, particularly glibenclamide, bind the SUR1 subunit and stabilize the channel in a closed state, thereby restoring insulin secretion. Early initiation of sulfonylurea therapy has been shown to improve both glycaemic control and neurodevelopmental outcomes [12,13], with reported median effective doses of 0.48 mg/kg/day (range, 0.017-2.6 mg/kg/day) [14,15]. Treatment is generally well tolerated and markedly reduces the burden of multiple daily insulin injections, while improving quality of life and cognitive outcomes [3]. This was clearly illustrated in our cohort, where infants with KATP channel mutations achieved excellent long-term metabolic control and normal neurodevelopment following transition to sulfonylurea therapy.

By contrast, neutral or poor outcomes were observed in children with syndromic or recessive forms of NDM. Wolcott-Rallison syndrome, caused by EIF2AK3 mutations, is characterized by early-onset diabetes and progressive multisystem involvement. The hepatic and extra-pancreatic complications observed in our patient are consistent with the natural history reported in other cohorts, where early-childhood mortality remains high [16]. These findings highlight the limitations of current therapeutic strategies when pancreatic dysfunction occurs as part of a broader systemic disorder, and support guideline recommendations emphasizing multidisciplinary management and early supportive care [3].

Finally, our cohort underscores the strong association between NDM and extra-pancreatic manifestations, including enteropathy, cytopenias, and autoimmune liver disease. These phenotypes are characteristic of immune dysregulation syndromes, such as IPEX and CASP10-associated ALPS, in which monogenic defects in immune regulation lead to early-onset diabetes accompanied by severe autoimmune and inflammatory complications. This observation reinforces ISPAD recommendations, advocating for broad genetic testing strategies extending beyond classical NDM genes, particularly in infants presenting with multisystem involvement or atypical clinical courses.

Finally, our experience highlights persistent health system challenges in resource-limited settings, where delayed referral, restricted access to comprehensive genetic testing, and socioeconomic barriers contribute to diagnostic and therapeutic delays. These constraints are not unique to our center and have been reported in global surveys of clinical practice, which emphasize substantial disparities in the availability of NGS and specialized care for monogenic diabetes across different regions. Addressing these gaps requires improved access to molecular diagnostics and the development of structured, multidisciplinary care pathways to ensure timely diagnosis and optimal management for all infants with NDM.

In summary, genetic testing plays a central role in the management of neonatal diabetes by enabling targeted therapy, refining prognosis, and facilitating accurate genetic counseling. Early identification of pathogenic variants allows for personalized treatment strategies, optimizing both metabolic control and neurodevelopmental outcomes. These findings reinforce the importance of integrating molecular diagnostics into routine clinical care for infants presenting with diabetes before six months of age.

Despite the valuable insights provided by this case series into the clinical and genetic heterogeneity of neonatal diabetes, several limitations must be acknowledged. The small sample size and single-center design limit the generalizability of our findings. Additionally, genetic testing could not be performed in all patients due to financial and technical constraints, potentially leading to underestimation of certain molecular etiologies. Long-term follow-up was incomplete for some patients, particularly those lost to follow-up after initial clinical improvement, and the retrospective nature of the study may have introduced selection and information biases. Nevertheless, this series offers meaningful insights into the spectrum of neonatal diabetes in a resource-limited setting and underscores the critical role of early genetic diagnosis in improving patient outcomes.

## Conclusions

NDM, whether transient or permanent, is a rare but informative monogenic condition that offers critical insights into the mechanisms of β-cell dysfunction and glucose homeostasis. It's pronounced genetic heterogeneity reinforces the essential role of insulin in fetal growth and early metabolic adaptation and highlights the importance of timely recognition to prevent metabolic decompensation and support adequate nutritional and neurodevelopmental outcomes.

In this retrospective descriptive case series, we documented substantial clinical and genotypic variability among affected infants. This variability reflects the broader spectrum reported in international cohorts. Our findings support the clinical utility of molecular diagnosis in neonatal diabetes, as genetic confirmation enabled tailored therapy in infants with ABCC8 mutations, clarified prognosis in syndromic forms, and guided genetic counseling. Although conducted in a resource-limited setting, our data indicate that early clinical suspicion combined with genotype-directed management can meaningfully improve outcomes. These observations underscore the need to expand access to genetic testing and ensure long-term follow-up, particularly given the potential for relapse in transient forms.
